# Context effects on the perception of saturation of fruit colors in still-life paintings

**DOI:** 10.1167/jov.23.13.8

**Published:** 2023-11-16

**Authors:** Matteo Toscani, Paulina Wolf, Karl R. Gegenfurtner, Doris I. Braun

**Affiliations:** 1 Psychology Department, Giessen University, Giessen, Germany; 2 Psychology Department, Bournemouth University, Poole, UK

**Keywords:** color, color saturation, still-life paintings, context effects, chromatic contrast

## Abstract

Still-life painters, especially of the so-called Golden Age (17th century) in the Netherlands, are famous for their masterful techniques of rendering reality. Their amazing abilities to depict different material properties of fruits and flowers are stunning. But how important are these careful arrangements of different objects for the perception of an individual item? Is the perceived color saturation of a single fruit influenced by its surrounding context? We selected fruits in still-life paintings as stimuli to investigate whether and how perceived saturations of fruits were affected by their original contexts. In our study, we focused especially on effects of five context properties: complementary colors, chromatic and luminance contrast, object overlap, and surround variance. Six fruit varieties depicted in high-quality digital reproductions of 48 classic and eight varieties in 64 more recent, modern still-life paintings were selected. In a single trial, eight images of fruits of the same variety appeared on a neutral gray background; half were single fruit cutouts, and the other half were the same fruits embedded in their circular contexts. Fifteen participants ranked all eight images according to perceived color saturations of the fruits. Saturation ratings showed a high agreement of 77%. Surrounding contexts led to an increase in perceived saturation of central fruits. This effect was mainly driven by object overlap, the presence of the central fruit type also in the context, and surround variance. Chroma contrast between fruits and contexts decreased saturation significantly. No significant context effects were found for complementary colors or luminance contrast. Our results show that in paintings, many of the cues that are usually experimentally isolated occur in interesting combinations and lead to an increase in perceived saturation that makes fruit objects more appealing and convincing.

## Introduction

During the Early Middle Ages, artistic painting techniques to depict people, scenes, landscapes, and objects and their different materials developed tremendously in Europe. Painters of the “Dutch Golden Age”^1^ around the 17th century are famous for their exquisite skills to create highly realistic scenes and lifelike appearances (*trompe-l’œil*) of people, animals, objects, and fabrics (Di Cicco, van Zuijlen, Wijntjes, & Pont, 2021; Wiersma, 2020). Craftsmanship, increasing knowledge about techniques, optical devices, and the application of oil as a painting medium instead of egg tempera, in addition to the use of layers and glazes, led to incredible convincing masterpieces like still-life paintings of Pieter Claesz, Willem Kalf, Willem Heda, and Jan Davidsz De Heem (Di Cicco, 2022; Di Cicco, Wiersma, Wijntjes, & Pont, 2020; Di Cicco, Wijntjes, & Pont, 2019; van Zuijlen, Pont, & Wijntjes, 2020; Smith, 2016). Today, it is hard to imagine how artists of that time period solved—especially when painting a still life—the problem of reproducing the complex patterns of reflections of light falling on complex arrangements of translucent wine glasses, metal cans and plates, and various materials of perishable goods like flowers, fruits, pastry, meat, poultry, or fish presented on various surfaces and fabrics on a canvas (Di Cicco et al., 2021). Artists did not necessarily paint an object in a way that it elicited the same retinal image as the physical one but appropriate enough to “fit” to visual perception (Cavanagh, 2005; van Zuijlen, Lin, Bala, Pont, & Wijntjes, 2021). Painters seemed to have used their experience, technical skills, and knowledge about visual perception to tackle different perceptual mechanisms that made the appearance of painted objects more convincing and that increased the intensity and vividness of their colors (Cavanagh, Chao, & Wang, 2008; Conway, 2012, Conway, 2022; Di Cicco, 2022; Di Cicco et al., 2019; Di Cicco et al., 2020). But in which way do context colors matter for the perception of central objects?

The palette of paint colors available during the “Golden Age” consisted mainly of mineral pigments like red and yellow ochre, umber, lime white, and vermilion; expensive grounded gemstones like lapis lazuli, jade, garnet, or malachite; crushed coal or bones; and plant extracts like madder lake or indigo (Barnett, Miller, & Pearce, 2006; Berrie, 2007). Some paint colors were quite toxic, such as lead white or vermilion, and some were light sensitive and changed or faded over time so that the former brilliance and colorfulness of the original painting was lost. However, the use of oil as a medium for paint colors, the technique to apply several layers of transparent diluted paints over opaquer colors and to glaze the surface, increased color intensities and saturation (Di Cicco et al., 2020). High demands for cheap, vibrant, but stabile colorants, especially for dye manufactures, fueled chemical research that finally allowed the commercial production of synthetic colors. In the 19th century, the range of colors for artists was enlarged by new brilliant, lightfast synthetic paint colors often based on chrome and cadmium for much lower costs (Barnett et al., 2006; Berrie, 2007; Conway, 2012). Besides the use of different paint colors, the paint style also became more diverse in 19th to 21st centuries. Representations of objects were often less realistic, paint colors were more saturated, and chromatic contrasts between objects and context were stronger (Ball, 2003; Conway, 2022; Gage, 1999). So far, it is unclear whether these factors play a role in the perception of saturation.

Color perception of an object is highly dependent on the context and cannot be described only based on its local properties alone. Here we wanted to know whether the techniques of painters, as well as the choice of colors and contrasts for the surrounding context of single objects, would manipulate our perception of color, especially its saturation. Saturation is probably the aspect of color perception studied the least (but see Hedjar, Toscani, & Gegenfurtner, 2023; Schiller & Gegenfurtner, 2016; Schiller, Valsecchi, & Gegenfurtner, 2018; Skelton & Franklin, 2020; Witzel, 2018). Colorimetrically, saturation is defined in the CIELAB color space as the ratio of chroma to lightness (C*/L*). When looking at natural, three-dimensional objects, shading leads to variations in both chroma and lightness across the object's surface; their ratio, however, remains constant. It is still unclear which properties of a visual scene contribute to the percept of saturation of single objects within a scene, but there seems to be good agreement between different observers in judging saturation, and natural scenes perception tends to be well predicted by standard measures of saturation (Schiller & Gegenfurtner, 2016). The hue of individual fruit objects is relatively uniform and stable (Ennis, Schiller, Toscani, & Gegenfurtner, 2018), but saturation can vary substantially between exemplars and serves as a fine-grained indicator for their ripeness (Mollon, 1995; Regan et al., 2001).

It has been known for a long time that the perception of saturation of a single chromatic patch depends also on its immediate surround. Already back in the 19th century, Chevreul (1861) described an increase of perceived color saturation of a central patch by the presence of complementary colors in its surround. These context effects were generalized to contrast gain, where the contrast of a central texture appears stronger in a low-contrast surround than in a high-contrast surround (Chubb, Sperling, & Solomon, 1989). Singer and D'Zmura (1994) showed that this phenomenon also holds for chromatic stimuli. Brown and MacLeod (1997) described a particularly interesting version of this phenomenon, the so-called gamut expansion. In their experiments, participants were asked to adjust according to the colors of six reference patches on a gray background the colors of six test patches presented in two surrounds with identical space-averaged luminance and chromaticity but different variance: One was a high-contrast, richly colored mosaic surround, and the other was another uniform gray surround. The results of the adjustments revealed that color appearance depended not only on the mean color of the surround but also on the variance and distribution of colors in the surround. For high-contrast colorful mosaics, participants adjusted the colors of the patches to much higher chromatic contrasts to compensate for the desaturating effect of the chromatic surround compared to the color adjustments for patches presented against uniform low-contrast gray surrounds. Further investigations concerning the critical conditions for the gamut expansion revealed that the effect was strongest when the central patch and its surround were isoluminant so that the luminance contrast was very low (Faul, Ekroll, & Wendt, 2008). It persisted when a variegated achromatic surround or a surround with high-luminance contrasts was used, and it was strongly attenuated when a black line separated the central patch from the surround or when the chromatic or luminance contrast was increased.

Our aim was to determine the influence of context on perceived color saturation of central fruit objects in still-life paintings. Why fruit stimuli of still-life paintings? One reason is that painters were/are very good observers and craftsmen, trained to paint all kinds of objects and materials lifelike and to use strategies to enhance perceptual effects. This is especially true for the still-life paintings of artists around the time period of the “Golden Age.” The realistic appearance of fruits matters for color perception as shown for color memory (Olkkonen, Hansen, & Gegenfurtner, 2008). The large variability of still-life paintings with respect to fruit objects and the surrounding arrangements allows one to select stimuli with very different image properties that may affect the perception of color saturation. The comparison of the perceived saturation of a single fruit with or without its painted immediate context may provide insights into compositional aspects, as well as the choice of colors and contrasts for the context to modify the perception of saturation. In particular, we investigated the impact of complementary colors, chromatic or luminance contrast, color variance, and object overlap of the immediate context surrounding a single fruit. To compare the effects of different styles and paint colors, we selected fruits of still-life paintings of two time periods, the 17th to 18th and 19th to 21st centuries.

## Methods

### Stimuli

**Figure 1.**
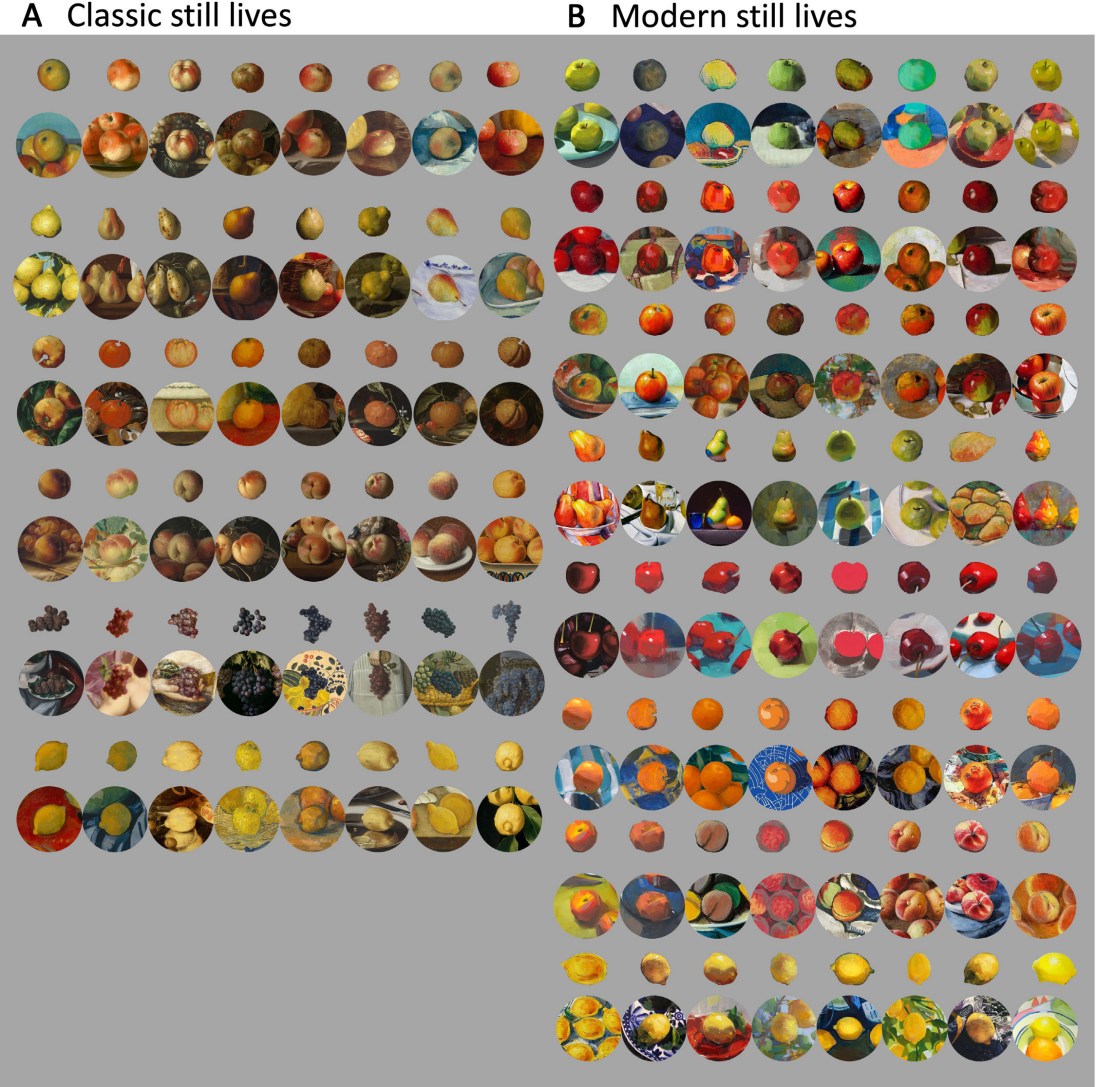
All stimuli. Fruits with and without their context selected from (A) classic (17th–18th centuries) and (B) modern still-life paintings (19th–21st centuries). Fruit categories are shown in six (classic) and eight (contemporary) separate rows (see Methods).

From the database of high-quality digital reproductions of nine online galleries of the Netherlands, Sweden, Spain, and the United States (van Zuijlen et al., 2021), 48 still-life paintings from the 17th to 18th centuries were used for our selection of fruit stimuli and their contexts, shown in Figure 1 (left side). From digital images of more recent and contemporary paintings (19th–21st centuries), 64 fruit stimuli and their contexts were selected. Their images were mainly taken from Wiki Art (see Figure 1, right side). For our experiment, we individuated single fruit depictions as test objects by making cutouts of the fruit only and by circular cutouts of the surrounding context together with the fruit in the center. Test stimuli belonged to different fruit categories. For the 17th to 18th centuries, six different fruits (apples, pears, oranges, peaches, grapes, and lemons) were tested. For the 19th- to 21st-century paintings, eight different fruit categories (green, red, and red-green apples; pears; cherries; oranges; peaches; and lemons) were presented.

**Figure 2.**
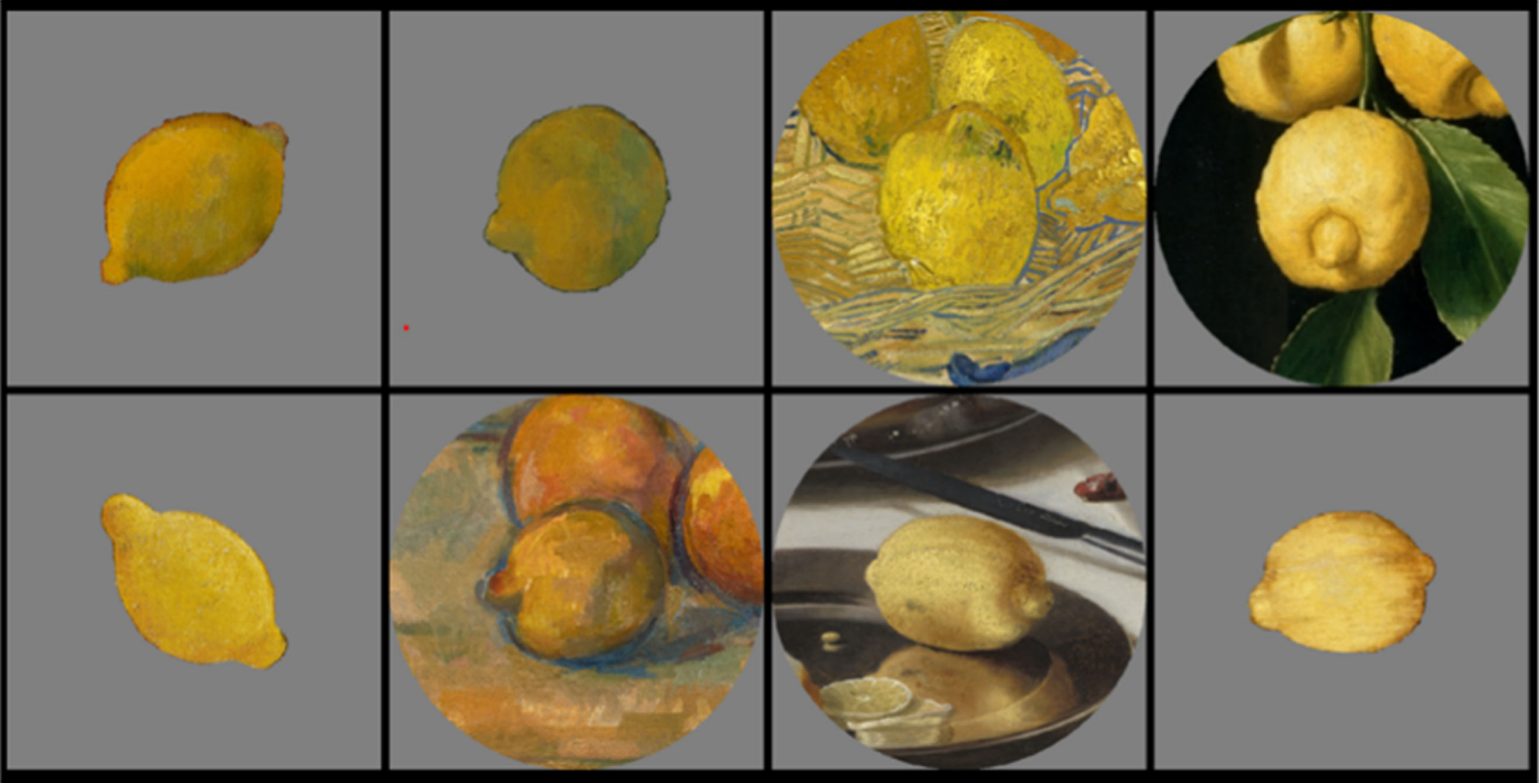
Example display of a single trial of the ranking experiment. Eight fruit stimuli were presented in a 2 × 4 arrangement on a gray background. In each trial, all fruits belonged to the same category and time period. In this example, all eight lemons were taken from paintings from 17th to 18th centuries; half of them were cutouts and presented without context, and the other half appeared in the centers of circular cutouts of their original contexts. In each trial, participants selected sequentially the most saturated fruit of all via mouse click and the chosen fruit disappeared.

For display, single fruit depictions were scaled to reach approximately 10 degrees of visual angle (dva), and their original circular contexts containing the same fruits in the center were scaled to 18 dva. In each single trial, eight fruit stimuli belonging to the same fruit category and time period were presented in a 2 × 4 arrangement on a neutral gray background. Half of the fruits were presented without and the other half with their context on a neutral gray background. The size of the four stimuli in each of the two rows was scaled to cover most of the horizontal dimension of the screen (see Figure 2). Fruit stimuli and their positions within the 2 × 4 arrangements were determined at random and changed with each repetition.

### Participants

Fifteen volunteers (8 males, average age: 26.87 years, *SD*: 10.8, range: 21–53 years) participated. Ten participants were psychology students of Giessen University, and others were friends or family members. All participants were naive to the purpose of the experiment. They were either reimbursed or got course credit for their participation. Before the start of the experiment, the color vision of all participants was tested with the 24-plate edition of the Ishihara test for color deficiencies (Ishihara, 2018). Then, a short introduction of the task was given, and after 3 minutes of dark adaptation, practice trials were run before the experiment started. All procedures were approved by the local ethics committee (Giessen University LEK 2013-0018) and in accord with the principles of the Declaration of Helsinki.

### Procedure

The experiment was conducted in a room with black painted walls and black blinds in front of the windows. During the experiment, lights were turned off, so that the room was only illuminated by the experimental computer monitor. Each participant sat with their head stabilized on a chinrest 38 cm away from a 22-in. computer monitor screen (Eizo CG245W 10-Bit LCD monitor; Eizo Nanao Corporation, Hakusan, Ishikawa, Japan) that was controlled by a Dell Precision T1700 Computer (Dell Inc., Round Rock, TX, USA). The effective monitor size was 1920 × 1200 pixels (71.42 × 44.64 dva). The monitor color primaries were R = (0.690, 0.305, 41.0), G = (0.212, 0.717, 103.8), and B = (0.149, 0.044, 12.87); D65 was set as the white point, and gamma was 2.2. Stimulus presentation was controlled using the Psychotoolbox (Kleiner, Brainard, & Pelli, 2007) and MATLAB, Version 2019b (The MathWorks Inc., Natick, MA, USA).

### Task

Participants were asked to sequentially “pick the fruit that appears the most saturated.” We told them to pick the object that appeared most “intensely colored” and otherwise followed the procedures established in Schiller et al. (2018). They indicated their choice by moving the computer mouse cursor to the chosen object and a left mouse click. The selected object disappeared and the participant continued to pick again the most saturated one until a single object was left in the display. Then the next trail started with eight different fruits of a different category. Each display included fruits from only one time period and one of the six fruit categories chosen for the 17th- to 18th-century paintings or of the eight fruit categories for the 19th- to 21st-century paintings (see Figure 1). Each combination of fruit category was repeated five times, for a total of 70 trials, in a randomized manner. Before each session, the task was explained with three practice trials consisting of three target displays generated with the same criteria as for the experimental trials (i.e., the same fruit category and time period). Practice trials allowed to familiarize participants with the ranking task procedure and to answer upcoming questions. Practice trials were excluded from the analyses. Each participant ranked 112 fruit stimuli.

### Ranking analysis

We extracted the *mean rank* position for each stimulus and computed the average *rank difference* between two conditions: fruit stimuli presented as cutouts without context on the gray background and fruit stimuli surrounded by their original contexts. Additionally, we determined the probability of each stimulus to be ranked as more saturated than the others (*probability to be more saturated*). We expressed the measure of the probability of each fruit to be perceived as more saturated when presented with its context compared to its probability when presented as a cutout as the *odds ratio*.

#### Mean rank

The eight fruit stimuli presented in each single trial were ranked in order of the participant's selection of perceived saturation from 1 (highest) to 8 (lowest). First, the rank value was inverted so that higher numbers corresponded to stronger perceived saturation. Then rank positions of each stimulus were averaged across trials, yielding an average rank for each of the 112 fruit stimuli per participant.

#### Rank difference

We subtracted the average rank of each fruit without context from the average rank of the same fruit presented with context.

#### Probability to be perceived as more saturated

We estimated the probability of each fruit to be perceived as more saturated than the other fruits shown in each trial. To do so, we converted for each trial rankings into paired comparisons, separately for each fruit stimulus with and without context. For each trial, four fruits were shown as cutouts and four fruits together with their contexts. For each group of four, and for each of the six pairs they yield, we determined based on the average ranking which fruit of each pair was perceived as more saturated. From the paired comparisons, we computed the probability of each fruit to be perceived as more saturated than others as the proportion of the pairs in which that fruit was ranked higher in the pair over all pairs in which the fruit was presented.

#### Odds ratio

We computed the odds ratio as follows:

$$\begin{array}{c} odds\;ratio=\frac{p_{s}}{1-p_{s}}/\frac{p_{ns}}{1-p_{ns}} \end{array}$$

with *p_s_* being the probability of being ranked higher than any other painting when presented with surround and *p_ns_* without surround. The odds ratio measures how much more likely a fruit is ranked higher than others when presented with the surround as compared to when presented without. If the odds ratio equals 1, chances are the same (i.e., the circular surround has no effect). If smaller than 1, the surround causes the stimuli to be ranked lower; if bigger than one, it leads to a percept of higher saturation. The odds ratios were first calculated for each observer separately and then averaged across observers.

### Color statistics

**Figure 3.**
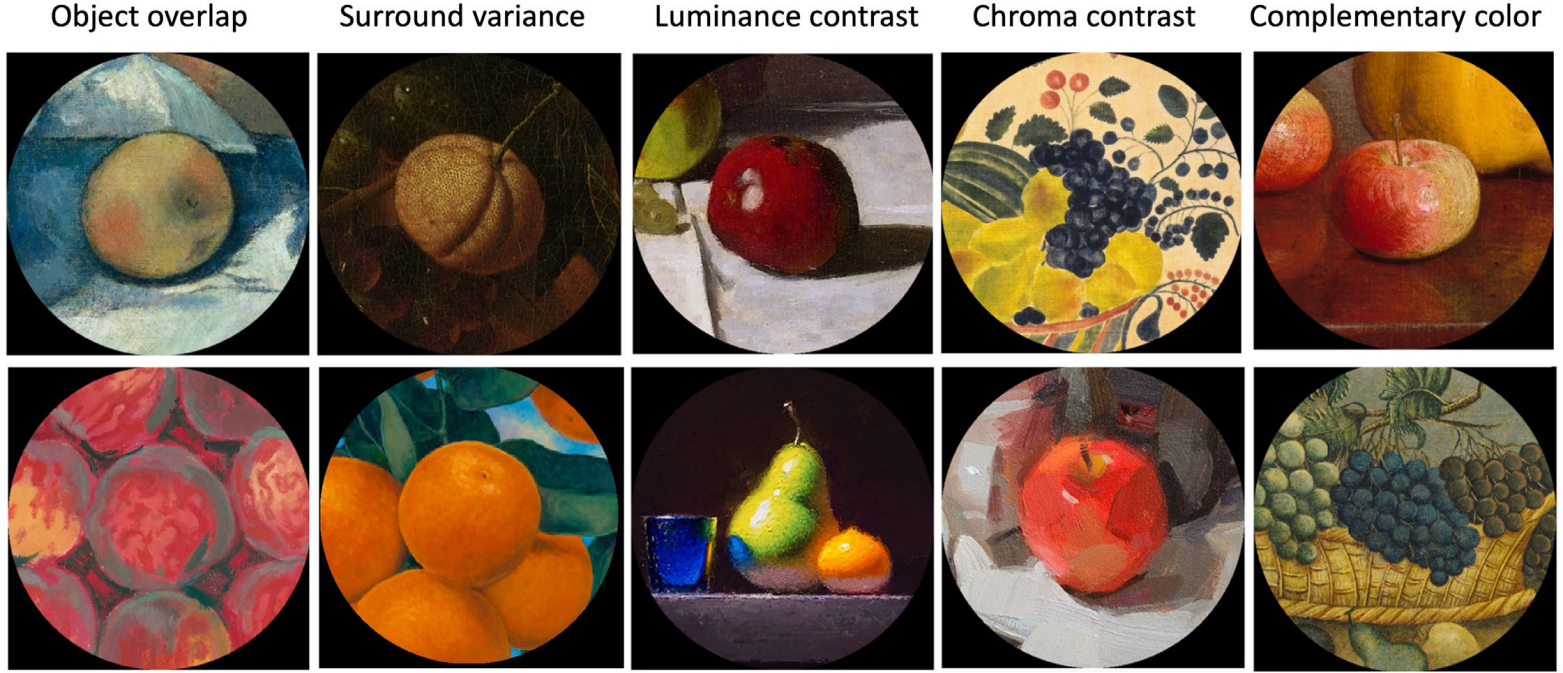
Examples of extreme scores of our five analyzed color and luminance statistics to illustrate their effects on perceived saturation. Examples for the minimum values are presented in the top row, and examples for the maximum values are in bottom row. Since for luminance contrast, the minimum is the same stimulus as for chroma contrast, we have selected the one with the next higher value.

For each stimulus, we investigated five color and luminance statistics to clarify their contributions to the appearance of saturation to each central fruit when surrounded by its context. Examples of cutouts with extreme values on these five statistics are shown in Figure 3. We compared the ranking values of the same fruits presented within their contexts to those of the same fruits as cutouts only. To do so, we related the odds ratio, our measure for context effects on the saturation ranking, with the following five measures computed after the color and luminance of each image pixel was expressed as L*, C*, and h in CIELAB color space (CIE, 1978). All analyses were performed with the color coordinates of the stimuli as displayed on the monitor. We also ran the analysis with the nominal sRGB values of the images, but this did not lead to any noticeable changes in figures or statistics.

#### Complementary color

With this measure, we aimed to capture chromatic induction (Chevreul, 1861) caused by the colors of the pixels of the surround on the central fruit object (i.e., the increased color contrast of the fruit in the complementary direction of the inducing surround) (Singer & D'Zmura, 1994). For this measure, the proportion of pixels in the surround with hues within the opposite half of the color circle with respect to the mean hue of the fruit was calculated. Chromatic induction may be used by artists to increase color “intensity” (e.g., Cernea, 2002). Figure 3 shows that the blue grapes surrounded by yellowish and greenish grapes and fruits and background objects scored maximally on complementary color.

#### Chroma contrast

We measured the contrast between the average chroma of the surrounding context and the average chroma of the central fruit as the ratio of the difference between the mean chroma of the fruit and the mean chroma of the context to the mean of these two. This measure was meant to capture how much induction effects can actually influence the color of the central fruit, since the higher the chroma of the fruit color, the less this color is subject to induction (e.g., Faul et al., 2008; Kirschmann, 1890). Also, induction effects are larger with a higher contrast of the inducer (Singer & D'Zmura, 1994). Figure 3 shows that a bright red apple with high chroma in a whitish surround with low chroma had the highest chroma contrast (bottom row), while chroma contrast was maximally negative for the picture of low-chroma blue grapes in a high-chroma yellowish background (top row).

#### Luminance contrast

We expressed the luminance (or brightness) contrast between the central fruits and their surrounding contexts as the ratio of the difference between the mean L* of each fruit and the mean L* of its context to the mean of these two. This measure could potentially contribute to explain an effect of the context on saturation because perceived saturation could be reduced within a dark environment (Pitt & Winter, 1974), and color patches are perceived as more saturated when the luminance contrast with their surroundings is reduced (Faul et al., 2008). Note that this measure is defined by signed contrast. It is zero for isoluminant contexts. It is negative when the surround is bright and the central fruit dark and positive when the central fruit is bright and the surround dark, as illustrated in Figure 3.

#### Surround variance of the context

This is a measure of color and brightness variance within the context, computed as the sum of the variances along the three dimensions of the CIELAB color space. It may affect the perceived saturation of the fruits in the center because perceived saturation is reduced in environments with a high color variance (Brown & MacLeod, 1997). Figure 3 illustrates that surround variance is high for very bright and structured contexts and low for uniformly dark contexts.

#### Object overlap

We used this measure to capture cases in which a fruit was surrounded by similar fruit objects of similar hues. In still-life paintings compositions, presentations of several fruits of the same kind in close proximity like a bowl of peaches or a basket of apples are quite common. We segmented the surrounding contexts to isolate fruits with a similar hue as the central fruit. Then we computed the object overlap by determining the proportion of pixels of the surrounding context occupied by such fruits. This measure could be relevant for perceived saturation, as color perception can be influenced by grouping phenomena (Xian & Shevell, 2004). Figure 3 illustrates that object overlap is maximal for a central fruit object surrounded by fruits of the same kind and low for a central fruit surrounded by objects of different kinds and colors.

### Effects of painting periods

**Figure 4.**
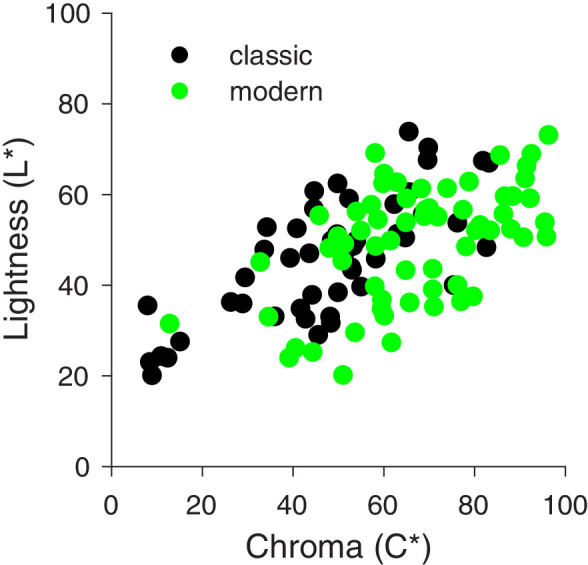
Scatterplot of CIELAB chroma C* versus lightness L* for all pixels of the central fruit objects for the classic (black circles) and modern (green circles) still-life paintings. Most of the modern paintings have much higher chroma values compared to the classic ones.

There are major differences between still-life paintings of the two time periods concerning not only the style but also the pigments used for the paints. Modern paint colors are based on pigments including cadmium, chrome, iron and cuprous oxide, molybdates, and various synthetic inorganic or organic components and fillers to increase their strength. The use of modern paint colors results in an increase of chroma, as can be seen in Figure 4. In the following, we will therefore compare the effects of the above chromatic statistics on perception of saturation for the two time periods.

## Results

**Figure 5.**
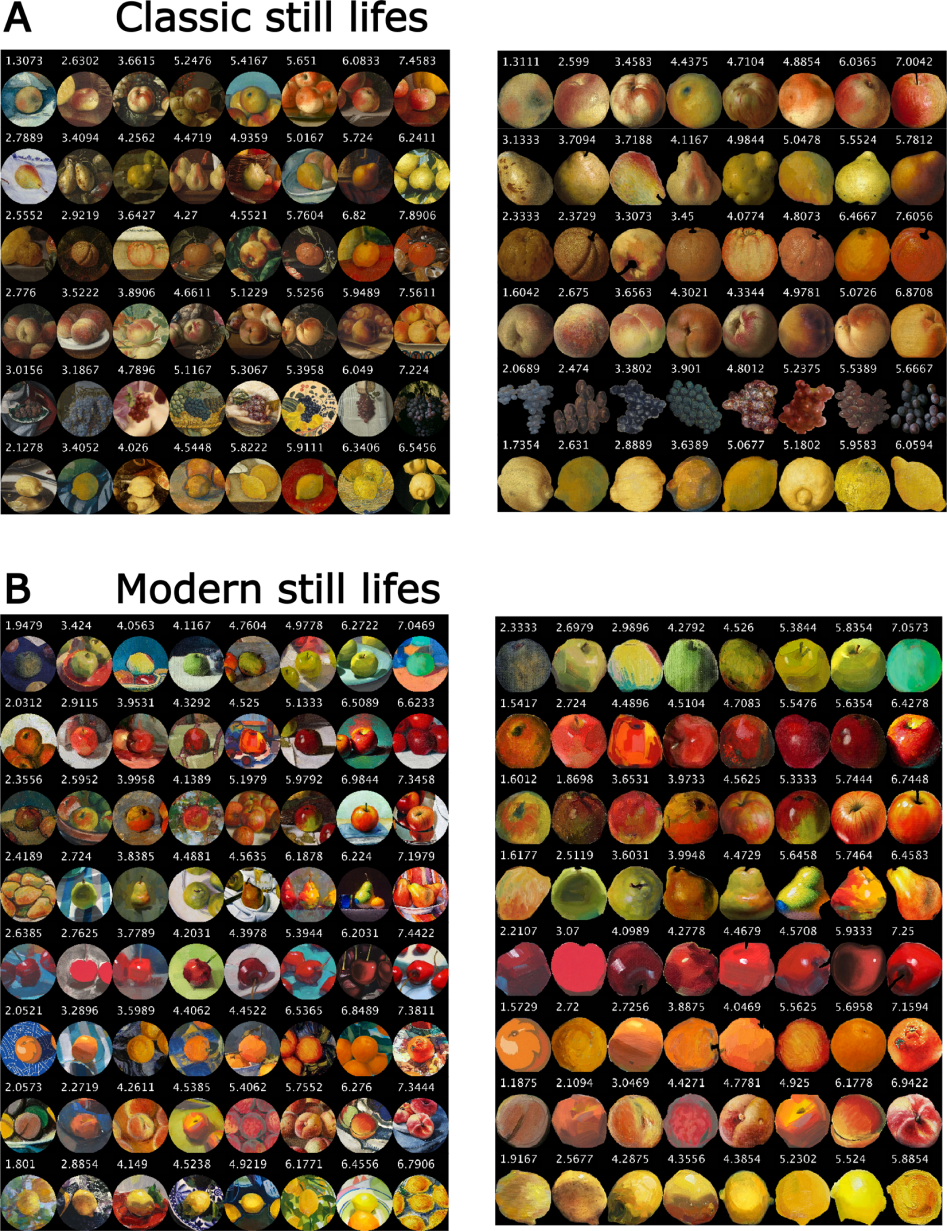
Observed rankings of all stimuli with and without context for (A) classic (17th–18th centuries) and (B) modern still-life paintings (19th–21st centuries). The fruits perceived to be most saturated are shown to the right of each row.

All our participants were able to rank the different fruit stimuli according to their perceived color saturation. Due to the randomized presentation of the fruits, not every participant was presented with all 112 fruits. Internal observer consistency for the 15 participants was calculated by determining the proportion of cases in which a participant's pairwise comparison for a fruit with and without context matched the response of the other participants. The consistency proportion was then averaged across all participants. Across all fruits, the internal observer consistency was 77%. Figure 5 shows all fruits with their rankings. The large range of the average rankings, between 1.3 and 7.8, is also an indicator for the high consistency of the observers. Figure 5, as does Figure 1, shows that there was relatively little variation of hue within each fruit category.

**Figure 6.**
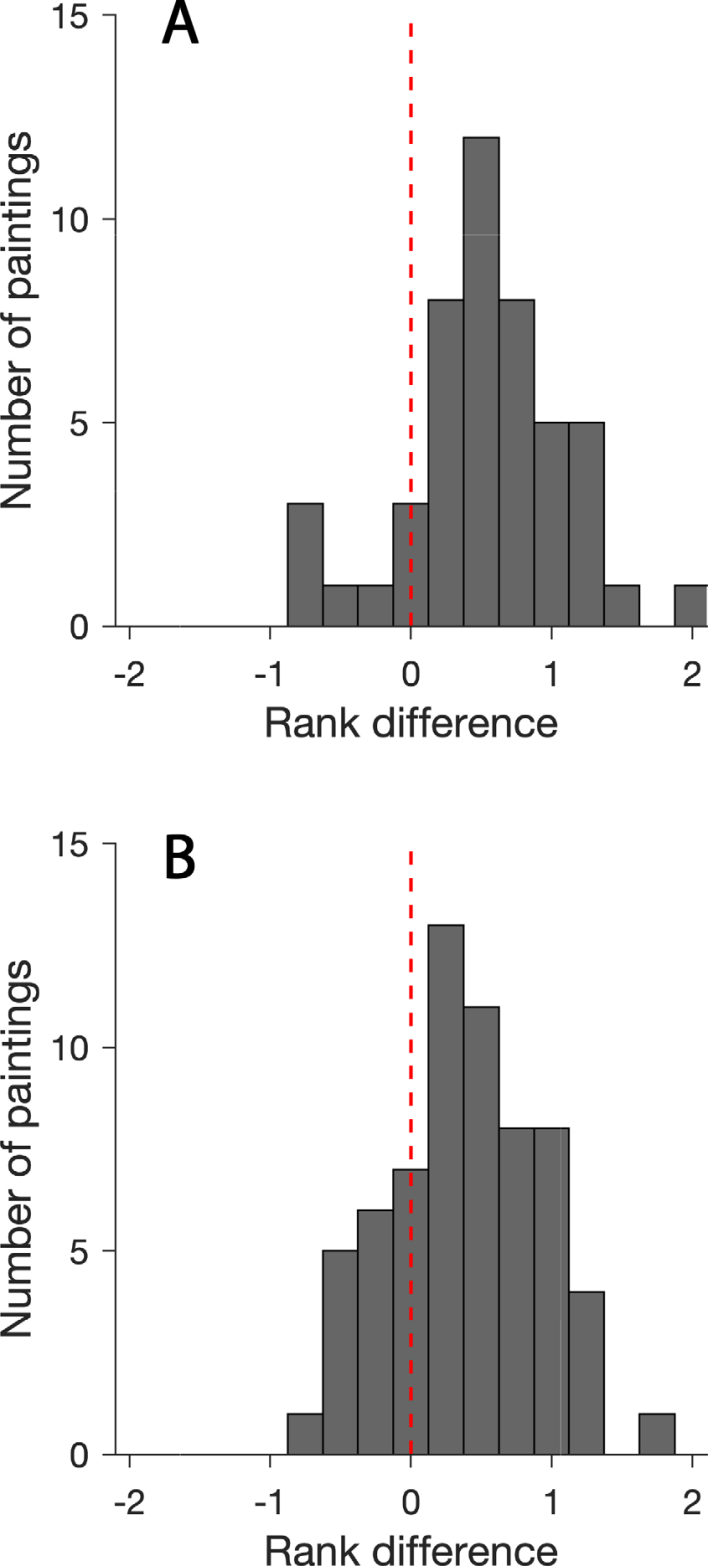
Histograms of the frequency of rank differences for (A) 48 fruit stimuli of the classic 17th- to 18th-century paintings and for (B) 64 fruit stimuli of the 19th- to 21st-century paintings. Rank differences (x-axis) were averaged across all participants. The rank difference for most stimuli is higher than zero, as indicated by the vertical broken red line. This result demonstrates that color saturation of fruits surrounded by their original contexts was rated higher in most cases.

When presented within their original contexts, most fruit stimuli had higher chances to be ranked as more saturated compared to their presentations as fruit cutouts. In Figure 6, relative frequencies of rank differences are presented separately for the two time periods (see for the classic Figure 6A and for the modern paintings Figure 6B).

**Figure 7.**
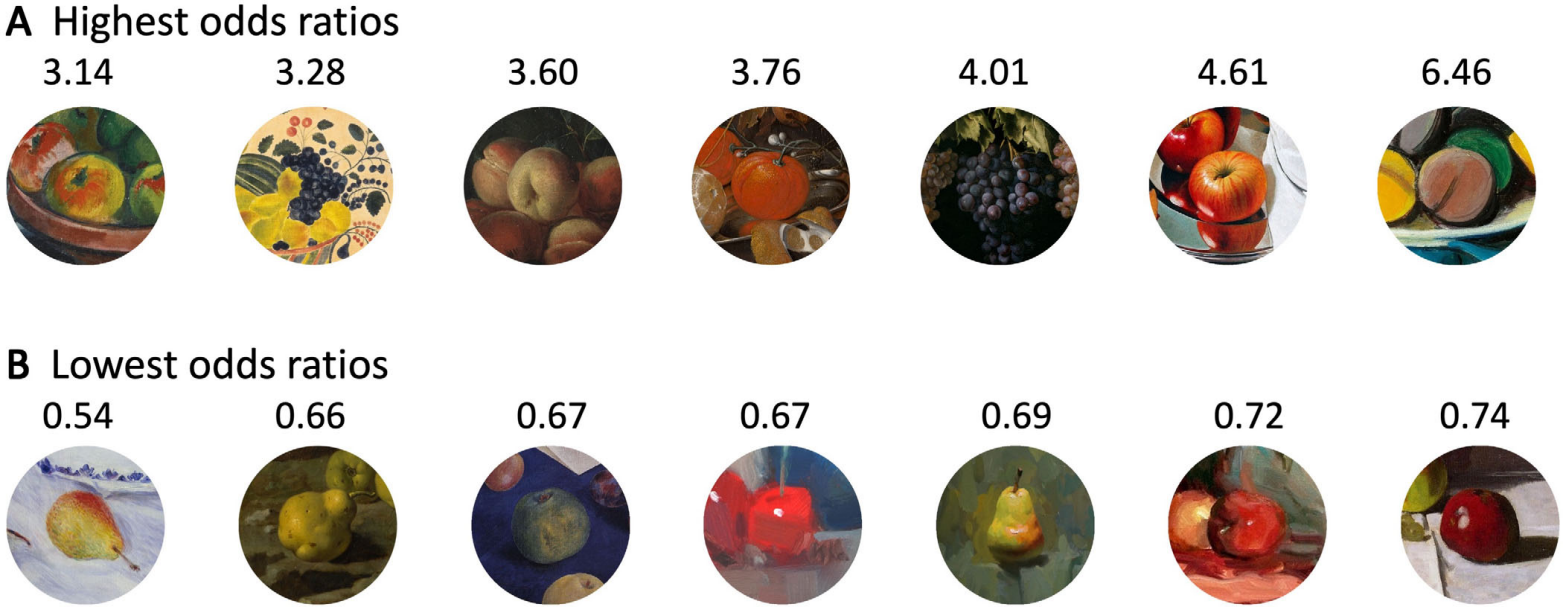
Examples of (A) high and (B) low odds ratios.

To investigate context effects on the perceived color saturation of the central fruit, we computed the odds ratio for each stimulus (see Methods). If the odds ratio equals 1, chances for rankings of perceived saturation are the same for fruits presented with or without context; if it is smaller than 1, saturation is ranked lower with context; and if it is bigger than 1, saturation is ranked higher with context. The *t*-tests against the null hypothesis (i.e., odds ratio equals 1) revealed significant positive context effects for perceived color saturation of fruit stimuli taken from classic (*t*(47) = 5.6296, *p* < 0.05) and modern still-life paintings (*t*(63) = 4.6362, *p* < 0.05). Figure 7 shows examples of images with the highest and lowest odds ratios. In some cases, fruits that were judged to be highly saturated without context became less saturated with context. Fruits of low perceived saturation in isolation became more saturated with context. These can be partly considered as regressions toward the mean.

### Regression analyses

In our analysis of context effects on perceived saturation of our fruit stimuli, we found that the contribution of the five chosen factors differed in an interesting and unexpected way that allowed a new explanation of context effects. Overall, it turned out that *chroma contrast, surround variance,* and *object overlap* had a strong impact on perceived color saturation. Neither the *proportion of complementary colors* nor *luminance contrast* changed the perceived color saturation of the central fruit significantly.

In the following section, we will first show linear regressions of the individual five predictors on the measured odds ratios and subsequently present a multiple regression analysis. Since every painting is a unique stimulus and differs in many aspects from all the others, general explanations of context effects may not always hold for each stimulus. To allow better comparisons and to account for the diversity of our stimuli, few selected stimuli are shown as small circular patches in scatterplots for the effects of the five context factors and the multiple regression model.

**Figure 8.**
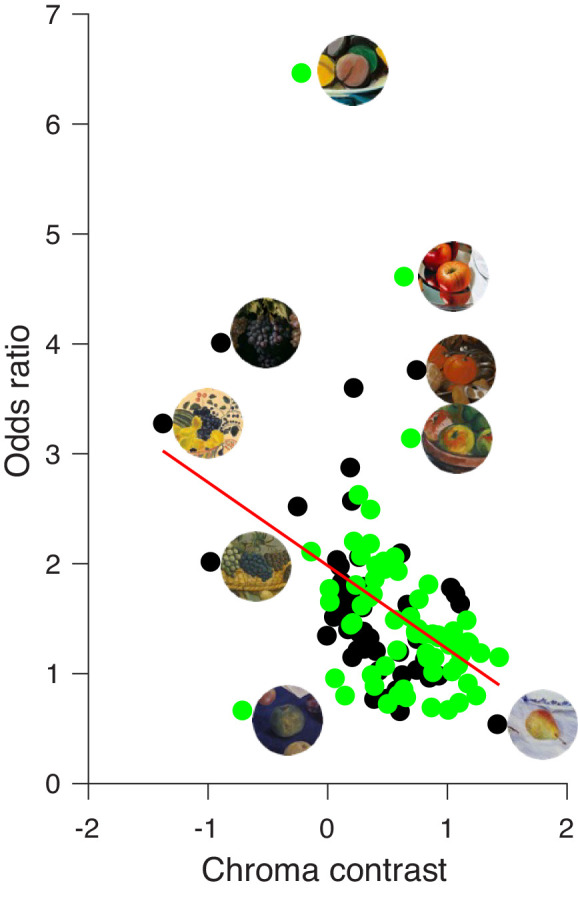
Relationship between chroma contrast (x-axis) and odds ratio (y-axis) for classic (black circles) and modern (green circles) paintings. When the odds ratio equals 1, the context has no effect; when it is smaller than 1, the context reduces; and when it is bigger than 1, it increases the perceived saturation of the central fruit compared to fruits without context. Few fruit stimuli with their contexts are shown next to their corresponding data points for illustration. The red line indicates the linear regression for all paintings.

The scatterplot of Figure 8 shows the relationship between chroma contrast (x-axis) and odds ratio (y-axis) for all fruit stimuli of both time periods. As in Figure 4, black symbols indicate the classic and green symbols the modern paintings. The result of the regression analysis states a significant association between chroma contrast and the odds ratio. For both of our two selections of fruit stimuli, this relationship is negative, *r* = −0.52, *p* = 0.001, for classic and *r* = −0.343, *p* = 0.006, for the modern stimuli. This indicates that context effects are reduced when the chroma of the fruit color increases and the chroma of the context decreases. There was a substantially higher (*t*(110) = 3.14, *p* < 0.005) chroma contrast in the modern paintings (62%) than in the classic paintings (34.8%). Still, the regression lines for both periods showed nearly complete overlap, and Figure 8 thus shows only the joint regression line for all paintings.

According to this result, colorful contexts increase the saturation of central colors with low chroma. This is at odds with predictions based on previous literature (D'Zmura & Lennie, 1986; Faul et al., 2008; Kirschmann, 1890). At least partially this relationship might be driven by fruit stimuli that are surrounded by complementary colors in the context. This may have increased their perceived saturation, for example, the dark blue grape surrounded by a yellow context in Figure 7A or the peach next to a green leaf in Figure 7B.

**Figure 9.**
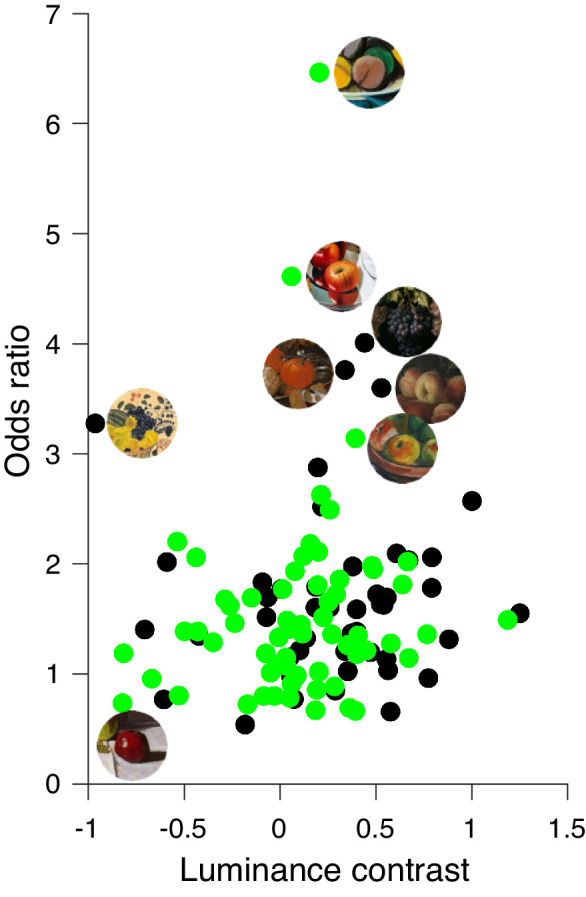
Relationship between luminance contrast (x-axis) and odds ratio (y-axis) for classic (black circles) and modern (green circles) paintings. Luminance contrast between context and fruit seems to have no significant influence on the perceived saturation of fruits of classic (*r* = 0.04, *p* = 0.785) and modern still-life paintings (*r* = 0.127, *p* = 0.317).

In Figure 9, the relationships between luminance contrast and odds ratio for both selections of fruit stimuli are plotted. There is no apparent relationship between luminance contrast and the odds ratio in both cases. In fact, a high odds ratio can be associated with high negative, high positive, or close to zero luminance contrast. This is incongruent with previous results (Faul et al., 2008) showing that color patches are perceived as more saturated when the luminance contrast with their surroundings is reduced. Taking the absolute luminance contrast does not lead to significant correlations either. There is a substantially higher (*t*(110) = 2.31, *p* < 0.005) luminance contrast in our stimuli of classic paintings (27.3%) than in stimuli of modern paintings (9.5%).

**Figure 10.**
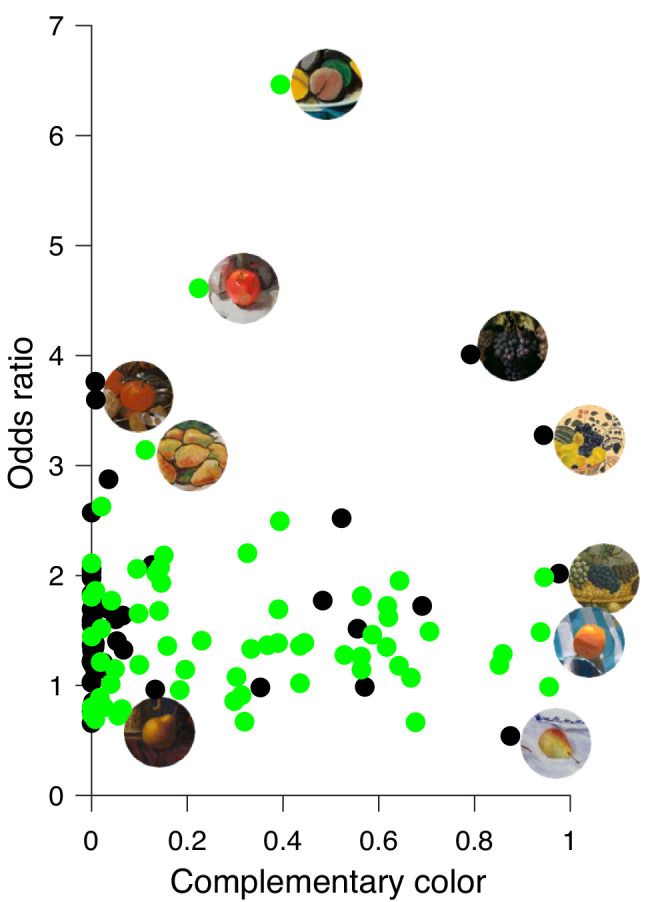
Relationship between complementary color (x-axis) and odds ratio (y-axis) for classic (black circles) and modern (green circles) paintings. Selected fruit stimuli with their contexts are shown next to their corresponding data points.

The scatterplot of Figure 10 shows the relationship between complementary colors and odds ratios for both painting periods. There is no significant correlation between complementary color and odds ratio either for stimuli of the classic (*r* = 0.237, *p* = 0.11) or of the modern paintings (*r* = −0.01, *p* = .936). Here we noticed that there was a substantially higher (*t*(110) = 2.95, *p* < 0.005) proportion of complementary color in the modern paintings (31.5%) than in the classic paintings (15.5%). Only few stimuli had higher amounts of complementary colors in their contexts and high odds ratios, and these stimuli seem to be the ones with particularly low chroma contrast (Figure 8).

**Figure 11.**
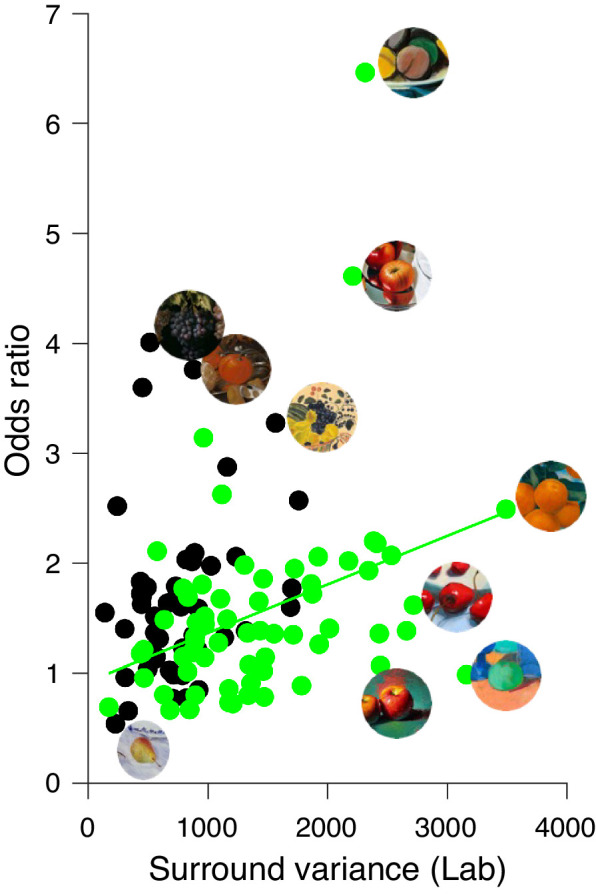
Relationship between surround variance of the context (x-axis) and odds ratio (y-axis) for classic (black circles) and modern (green circles) paintings. The green line shows the regression line for the modern paintings. Selected fruit stimuli with their contexts are shown next to their corresponding data points for select paintings.

The regression analysis revealed a significant positive relationship between surround variance of the context and odds ratio for fruits of modern paintings (Figure 11, green line, *r* = 0.353, *p* = 0.004) but not for the classic ones (*r* = 0.244, *p* = 0.095). This result indicates that a high surround variance in the context increases perceived saturation of the central object, and it differs from the finding of Brown and MacLeod (1997), who found that saturation is reduced in environments containing high color variance. However, one reason for the different effects of surround variance on color appearance may result from the difference in saturation of the targets used. Brown and MacLeod presented six grayish low-contrast rectangles embedded in two surrounds with different variances but with identical space-averaged luminance and chromaticity. Subjects adjusted the colors of the six grayish rectangles in the high-contrast richly colored surround to match the colors of the six rectangles on the uniform gray surround. In our experiment, subjects ranked naturally colored painted fruits presented as cutouts on a gray background or within their painted contexts. The surround variance of the contexts differed by a large degree for the two painting periods in addition to the saturation of the paint colors used. There was a substantially higher (*t*(110) = 5.81, *p* < 0.0001) surround variance in the modern paintings (1,421) than in the classic paintings (754).

**Figure 12.**
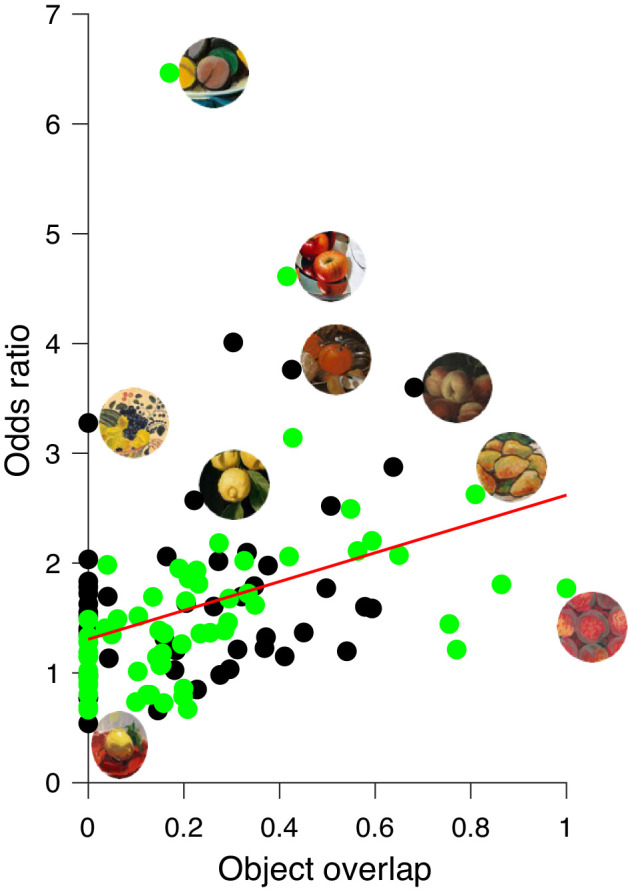
Relationship between object overlap (x-axis) and odds ratio (y-axis) for classic (black circles) and modern (green circles) paintings. The red line indicates the linear regression for all paintings. For both sets of stimuli, the relationship was positive and significant for the classic (*r* = 0.353, *p* = 0.014) and modern paintings (*r* = 0.35, *p* = 0.005). Few example stimuli are shown next to their data points.

The relationship between object overlap of the central of fruit and its context and the odds ratio turns out to be also positive and significant for both stimuli selected from classic and modern paintings, as revealed by the regression analysis (see Figure 12). Increasing object overlap goes along with an increase in odds ratio, for both the classic (*r* = 0.35, *p* < 0.05) and the modern (*r* = 0.35, *p* < 0.005) paintings. The regression lines lie on top of each other; therefore, we only plot the common regression line for all paintings. In our sample (see Figure 1), several stimuli of our selection of the 48 classic and of the 68 modern still-life paintings were depicted next to the central fruit parts of at least two of the same fruit category in the small circular cutouts of the original contexts. There was no significant difference (*t*(110) = 0.05, *p* > 0.5) in object overlap between classic and modern paintings with an overall average of 22.5%, but in modern paintings, similar fruits were often grouped much closer together. Therefore, similarities, in both color and shape, of the central fruit and surrounding context seem to be a good and well-known artistic strategy to enhance the percept of color saturation of central objects.

### Multiple regression

In addition to the individual regressions, we also used a multiple regression approach to assess the relationship between the five color and luminance statistics and the measured context effects on perceived saturation expressed by the odds ratio. We considered a regression model with all the two-way interaction terms and a simple model with no interactions. The more complex model failed to significantly explain more variance than the simpler version (*F*(15, 91) = 1.684, *p* = 0.068); therefore, we opted to not include interaction terms. A collinearity analysis revealed that the correlations between predictors were limited. The variance inflation factor computed for each predictor ranged between [1.05 1.24]. As a rule of thumb, values < 10 (Kutner, Nachtsheim, Neter, & Wasserman, 2004) or < 5 (Sheather, 2009) allowed for a safe interpretation of the applied regression analysis. Regression results for the simpler model are shown in Table 1.

**Table 1.** Regression results.

Color and luminance statistic	Regression coefficient	*SE*	Standardized coefficient	*t*	*p*
Surround variance*	2.719e-4	1.214e-4	0.219	2.240	0.027
Object overlap*	0.907	0.346	0.242	2.618	0.01
Complementary color	0.292	0.267	0.101	1.094	0.276
Luminance contrast	0.308	0.178	0.149	1.733	0.096
Chroma contrast*	–0.601	0.172	–0.332	–3.488	<0.001

* Indicates statistics with a significant effect on the odds ratio.

**Figure 13.**
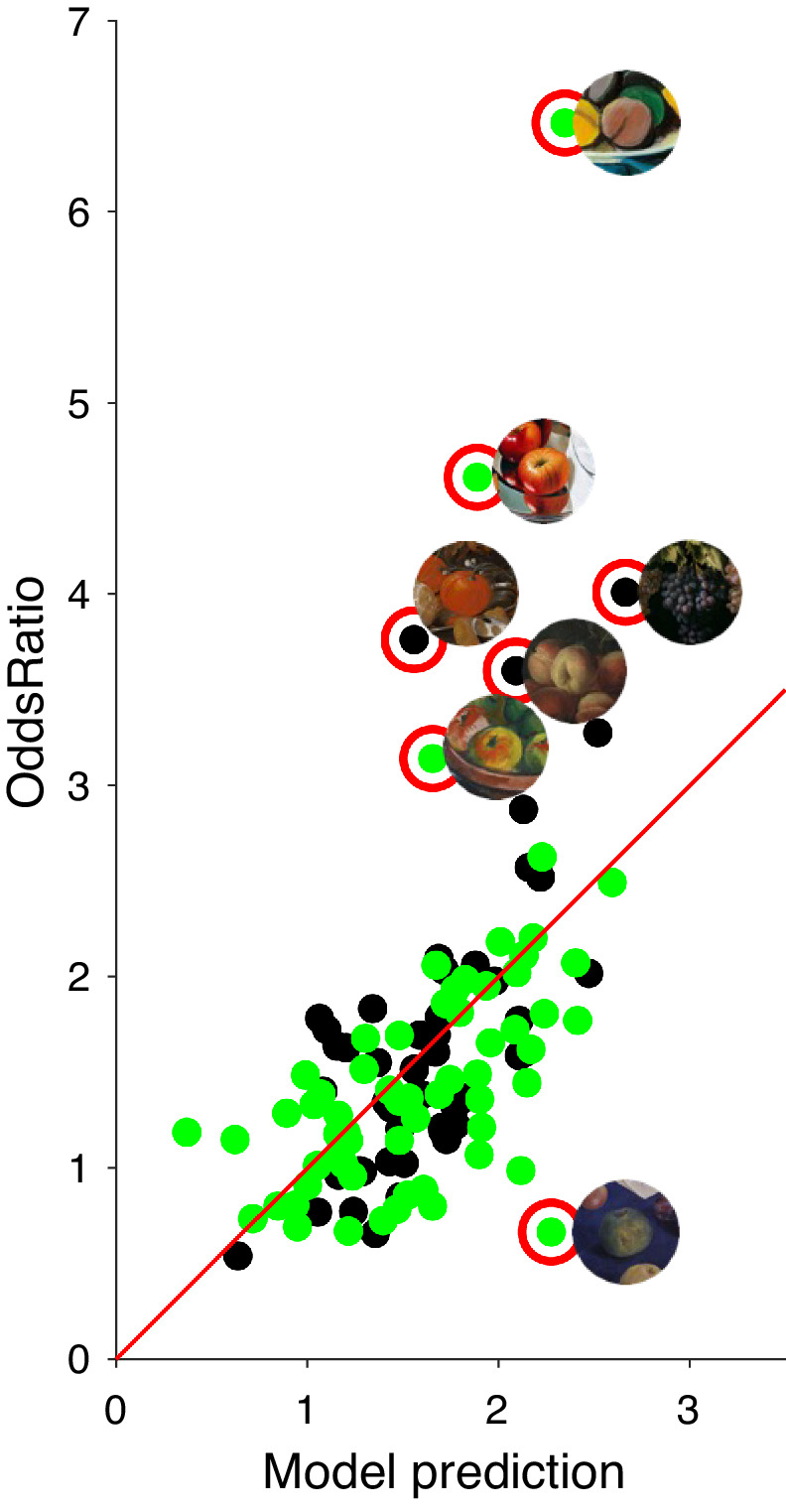
Relationship between the multiple regression model predictions (x-axis) and odds ratio (y-axis) for the 17th- to 18th-century (black symbols) and for the 19th- to 21st-century (green symbols) paintings. The seven stimuli deviating significantly from the multiple regression model are shown next to their corresponding data points. The red line indicates the linear regression for all paintings.

In Figure 13, the odds ratio is plotted as a function of the multiple regression model's predictions. The overall correlation is quite high. Pearson's *R* is 0.55 for both periods together, as shown in Figure 12. Separately, it is 0.65 for the classic paintings and 0.49 for the modern ones. All of these correlations are clearly significant (*p* < 0.005). The strongest contributions came from chroma contrast, object overlap, and surround variance, as was already shown in the individual regressions above. The images with the highest odds ratios were therefore characterized by negative chroma contrast, large surround variance, and large object overlap.

## Discussion

Still-life paintings are highly complex stimuli that allow studying the contribution of a variety of factors to perceived saturation of color. We extracted fruit stimuli and their original contexts from digitized images of still-life paintings from two different time periods (17th−18th and 19th–21st centuries) to investigate whether the presence of the original context surrounding a single fruit influenced its perceived color saturation. The high agreement of the saturation ratings demonstrated that participants could judge perceived color saturations of the fruit stimuli consistently. Compared to the same fruits shown as cutouts on a gray background, the presence of the original context increased the perceived saturation of the central fruit significantly. Regression analysis for the specific contribution of five different image statistics revealed that three factors exert a significant influence on perceived saturation of the color of the central fruit. Interestingly, the effects of these five image statistics were nearly identical for paintings from both periods, even though their values differed considerably between the time periods. Significant positive effects of high degrees of object overlap of context and central fruit on perceived color saturation were found for the selection of fruit stimuli of both time periods. This is also true but weaker for surround variance, while chroma contrast had a significant negative effect on saturation. For complementary colors, mainly present in the contexts of modern still-life paintings and for luminance contrasts, hardly any context effects on perceived color saturation of the central fruit were found. The increase in saturation resulting from the presence of similar objects in the context is a novel observation that might explain why in still-life paintings, arrangements of the same or similar fruits in close proximity like a basket or bowl of apples, grapes, lemons, or peaches are quite common.

For our stimuli, we used a database of high-quality digital images of classic still-life paintings depicting typical arrangements of fruits, food, and valuable household items like glasses, plates, silverware, and carafes. The main criteria for our selection of digital images of still-life paintings was that single fruits should not be covered by other items so that it was possible to cut out whole fruits. Intact fruit shapes were important for the comparison of perceived saturation of fruits presented as cutouts with that of fruits embedded in their original contexts. The comparison of the still-life paintings of the two time periods revealed that the context increased the perceived saturation for most stimuli. Complementary colors in the context were mainly present in the selection of our contemporary paintings and rare in classic still-life paintings. Effects of complementary colors are known (Singer & D'Zmura, 1994) and used by artists (Cernea, 2002). The finding that object overlap between context and central object has a strong positive effect on color saturation is new and interesting. It may explain why arrangements of similar fruits are so common in still-life paintings and why we found so many in our two samples. Artists know how to compose a pleasing painting and where to place a certain object in certain size and color in combination with others for a convincing impression of the whole composition.

A large object overlap does go along with a larger area covered by the same or similar colors as in the center, and the resulting increase in size could potentially explain the increase in perceived saturation, if colors of similar fruits in close proximity are processed as a whole. It is known that larger stimuli appear brighter (Gombos & Schanda, 2006; Ronchi, 2002; Withouck, Smet, & Hanselaer, 2015). Furthermore, it has been proposed that brighter stimuli appear more saturated (Schiller et al., 2018) and that more saturated stimuli look brighter (Fairchild, 2013; Kohlrausch, 1920). Finally, saturation and size are associated, as more saturated objects appear bigger (Ling & Hurlbert, 2004). To answer whether bigger objects appear more saturated (Davidoff, 1991), we performed pilot experiments and asked participants to match the color saturation of a three-dimensional matte graphic (Mitsuba) object (Todd & Norman, 2018) to that of the same fruit presented separately as cut out in a small and large size and with its context containing the same or similar fruits grouped together. The preliminary results collected with only five of our stimuli that had larger object overlaps showed, besides positive context effects, size effects on saturation. Higher saturation adjustments found for larger fruits seemed to point to a significant contribution of size to perceived saturation. However, size alone could not explain the full effect of object overlap. Since there was too much variation between the five paintings to allow firm conclusions, we are planning more extensive experiments to clarify this question in more detail.

So far, our experiment provides evidence that object overlap and surround variance in the context explain parts of positive context effects of perceived color saturation of the surrounded object. However, potentially other factors relevant have not been considered in our analyses so far. Different aspects of the color distributions may affect perception of saturation (e.g., lightness is driven by the intensity of the brightest regions of the object color rather than the mean) (Toscani, Valsecchi, & Gegenfurtner, 2013). Assuming that colors of groups of fruits are processed as an ensemble and that the integration process results in higher values (like for lightness), higher perceived saturation may occur just because with more elements, there are more chances that the relevant color statistic reaches a higher value. This could be addressed with specific experimental manipulations to dissociate the statistics of the central fruit and of those in the surrounding context.

Luminance contrasts between contexts and fruits seemed to have both positive and negative effects on saturation. It is known that perceived saturation of a patch can be reduced by a dark environment (Pitt & Winter, 1974) and that color patches are perceived as more saturated when the luminance contrast with their surroundings is reduced (Faul et al., 2008). We found mixed results for the few fruit stimuli that had a lower rating of perceived saturation when presented with context (odds ratio < 1): 8 stimuli out of 48 for the classic and 14 out of 64 for the modern stimuli. Two of these eight fruits of classic paintings were surrounded by a lighter context, three by a context of about equal lightness, and three by a darker, greenish brown context. Of the 14 stimuli of the modern paintings, seven of the fruits were apples, four green and three red. Five of the modern fruits had a lighter gray or lighter-colored context, five a medium and four a dark background.

One of the most unexpected findings of our study was a negative effect of chroma contrast on context effects. Stimuli with low central chroma and high surround chroma exhibit large odd ratios. While the low central chroma leads to a low ranking of the isolated fruit, results of previous experiments with simple stimuli (D'Zmura & Lennie, 1986; Faul et al., 2008; Kirschmann, 1890) would suggest that a high chroma surround should lead to even lower rankings. This was not the case in our study. High chroma surround often led to a massive increase in the perceived saturation of the central fruits, especially when paired with a high surround variance. A low chroma contrast also occurs for stimuli of fruits surrounded by similar fruits. This may partly cause the negative relationship between chroma contrast and context effects on saturation. Such a relationship is indeed unlikely to be authentic as it conflicts with previous findings (D'Zmura & Lennie, 1986; Faul et al., 2008; Kirschmann, 1890). However, chroma contrast may influence the effect of the context in case that center and context exhibit opponent colors, as previous findings are based on results with simultaneous contrast displays (D'Zmura & Lennie, 1986; Faul et al., 2008) and do not apply to the stimuli with similar fruits (with a similar hue) in the center and the context. In general, our results show that many of the cues that are usually tested experimentally in isolation occur in paintings in interesting combinations that lead to a variety of surprising effects.

Our regression analysis is only able to suggest candidate predictors, whereas proper assessment of their individual contributions for the percept of saturation of color would need image manipulations designed to single them out (Wiebel, Toscani, & Gegenfurtner, 2015). In general, our multiple regression model can make quite good predictions when the contribution of chroma contrast, object overlap, and surround variance is included. While we discussed the general characteristics of this model with respect to the five predictors above, it is interesting to look at those particular paintings that deviate most from the model. For the seven paintings singled out in Figure 13, it is worthwhile to take a closer look at Figures 5 and 7. For six of these, their odds ratios were much higher than predicted by the model (i.e., their contexts had a much bigger effect than predicted). For three of them (the two peaches and the red-green apple), the central fruits presented alone got the lowest saturation ratings, so there was ample opportunity for context to show its effect. The other two (orange and apple) already had very high saturation rankings without context but improved even further. In both cases, object overlap is present due to a similar fruit in the context.

Why would painters strive to make the fruits appear more saturated? Still-life paintings depicted the lifestyle of the wealthy middle class by presenting expensive tableware like glasses, plates, and luxurious food items and flower decoration. For painters, it was probably very important to create and paint very appealing and convincing still-life arrangements, especially of quite valuable items. This required presenting the different materials like glasses, silverware, and cloths as convincing as possible and to paint also the food items and flowers as fresh and desirable to increase their value. Although it is still unclear how judgments of saturation are achieved (Hedjar et al., 2023), for the ripeness of fruit, hue and saturation seem to be an important diagnostic tool for the degree of sugar content. While many fruits like apples or pears have a thick skin, which changes its color over time, some other fruits, like grapes or red currants or peeled and cut citrus fruits, are semitranslucent. Translucency and juiciness indicate their ripeness and freshness, but neither of them was correlated to color saturation (Di Cicco et al., 2020).

## Summary and conclusion

With stimuli taken from classic and contemporary still-life paintings, we investigated whether the surrounding contexts modify perceived color saturation of central fruits compared to isolated fruit cutouts and which of five different color and luminance statistics of the stimuli modifies perceived saturation in a significant way. We found that the original surrounding context has a positive effect on perceived color saturation of the central object in most cases. Hue similarity between central fruit and context has a significant positive effect on perceived saturation, while the induction effect of chroma decreases with a decrease of the surrounding chroma. Neither the proportion of complementary colors in the context, which were rare in classic still-life paintings, nor luminance contrast changed the perception of color saturation of the central fruit. In our data set, object overlap is typically produced by close arrangements of similar fruits. The effect of object overlap can potentially be explained, at least in part, by the effect of size on perceived saturation, assuming that groups of similar fruits are grouped and processed together. These results suggest that similar objects in the context, possibly due to a similarity not only in hue but also in spatial frequency (Zaidi, Yoshimi, Flanigan, & Canova, 1992), let objects in the center appear more saturated and presumably also more appealing and realistic.

## Footnotes

1 It is important to note that the term *Dutch Golden Age* ignores the historical fact that the economic prosperity of the Dutch Republic came also at the price of poverty, forced labor, colonialism, and the transatlantic slave trade like that of other European maritime nations. Here we use the term to describe the artistic style of still-life painters during the time period of the 17th to 18th centuries.

## References

[bib1] Ball, P. (2003). *Bright earth: Art and the invention of color*. Chicago, IL: University of Chicago Press.

[bib2] Barnett, J. R., Miller, S., & Pearce, E. (2006). Colour and art: A brief history of pigments. *Optics & Laser Technology,* 38(4–6), 445–453.

[bib3] Berrie, B. H. (2007). *Artists' pigments: A handbook of their history and characteristics* (Vol. 4). Washington, DC: National Gallery of Art.

[bib4] Brown, R. O., & MacLeod, D. I. (1997). Color appearance depends on the variance of surround colors. *Current Biology,* 7(11), 844–849. 9382808 10.1016/s0960-9822(06)00372-1

[bib5] Cavanagh, P. (2005). The artist as neuroscientist. *Nature,* 434(7031), 301–307. 15772645 10.1038/434301a

[bib6] Cavanagh, P., Chao, J., & Wang, D. (2008). Reflections in art. *Spatial Vision,* 21(3), 261–270. 18534102 10.1163/156856808784532581 PMC2766568

[bib7] Cernea, P. (2002). The simultaneous contrast of the colors in Van Gogh paints. *Oftalmologia (Bucharest, Romania: 1990),* 55(4), 96–100. 12723187

[bib8] Chevreul, M. E. (1861). *Des couleurs et de leurs applications aux arts industriels à l'aide des cercles chromatiques*. Paris: Baillière.

[bib12] Chubb, C., Sperling, G., & Solomon, J. A. (1989). Texture interactions determine perceived contrast. *Proceedings of the National Academy of Sciences,* 86(23), 9631–9635. 10.1073/pnas.86.23.9631 PMC298552 2594791

[bib13] CIE (1978). Recommendations on uniform color spaces, color difference equations, psychometric color terms, Supplement 2 to CIE publication 15 (E1.3.1) 1971/(TC1.3). Central Bureau of the Commission Internationale de l'Éclairage (Vienna, Austria).

[bib14] Conway, B. R. (2012). Color consilience: Color through the lens of art practice, history, philosophy, and neuroscience. *Annals of the New York Academy of Sciences,* 1251(1), 77–94. 22429199 10.1111/j.1749-6632.2012.06470.x

[bib15] Conway, B. R. (2022). Vision and art. In *Oxford research encyclopedia of neuroscience*, 10.1093/acrefore/9780190264086.013.361.

[bib16] Davidoff, J. (1991). *Cognition through color*. Cambridge, MA: The MIT Press.

[bib17] Di Cicco, F. (2022). The legacy of Willem Beurs‒Bridging the gap between art and material perception. *Art & Perception,* 10(2), 111–136.

[bib18] Di Cicco, F., van Zuijlen, M. J., Wijntjes, M. W., & Pont, S. C. (2021). Soft like velvet and shiny like satin: Perceptual material signatures of fabrics depicted in 17th century paintings. *Journal of Vision,* 21(5), 1–22, 10.1167/jov.21.5.10. PMC8132013 33978685

[bib19] Di Cicco, F., Wiersma, L., Wijntjes, M., & Pont, S. (2020). Material properties and image cues for convincing grapes: The know-how of the 17th-century pictorial recipe by Willem Beurs. *Art Percept,* 8, 671–675.

[bib20] Di Cicco, F., Wijntjes, M. W., & Pont, S. C. (2019). Understanding gloss perception through the lens of art: Combining perception, image analysis, and painting recipes of 17th century painted grapes. *Journal of Vision,* 19(3), 1–15, 10.1167/19.3.7. 30897625

[bib21] D'zmura, M., & Lennie, P. (1986). Mechanisms of color constancy. *Journal of the Optical Society of America A,* 3(10), 1662–1672. 10.1364/josaa.3.001662 3772628

[bib22] Ennis, R., Schiller, F., Toscani, M., & Gegenfurtner, K. R. (2018). Hyperspectral database of fruits and vegetables. *Journal of the Optical Society of America A,* 35(4), B256–B266. 10.1364/JOSAA.35.00B256 29603941

[bib23] Fairchild, M. D. (2013). *Color appearance models*. New York, NY: John Wiley & Sons.

[bib24] Faul, F., Ekroll, V., & Wendt, G. (2008). Color appearance: The limited role of chromatic surround variance in the “gamut expansion effect”. *Journal of Vision,* 8(3), 30, 10.1167/8.3.30. 18484836

[bib25] Gage, J. (1999). *Color and culture: Practice and meaning from antiquity to abstraction*. Berkeley: University of California Press.

[bib26] Gombos, K., & Schanda, J. (2006). Interrelationship between size and brightness dimensions of appearance. In *CIE Expert Symposium on Visual Appearance*. University of Pannonia.

[bib27] Hedjar, L., Toscani, M., & Gegenfurtner, K. R. (2023). Perception of saturation in natural objects. *Journal of the Optical Society of America A,* 40(3), A190–A198. 10.1364/JOSAA.476874 37133037

[bib28] Ishihara, S. (2018). *Ishihara's tests for color deficiency*. 24 Plates Edition. Tokyo: Kanehara Trading.

[bib29] Kirschmann, A. (1890). *Ueber die quantitativen Verhältnisse des simultanen Helligkeits-und Farben-Contrastes*. Leipzig: Engelmann.

[bib30] Kleiner, M., Brainard, D., & Pelli, D. (2007). *What's new in Psychtoolbox-3?* Perception, 36 ECVP Abstract Supplement.

[bib31] Kohlrausch, A. (1920). Der Flimmerwert von Lichtmischungen. *Berichte über Die Gesamte Physiologie Und Experimentelle Pharmakologie,* 3, 589–591.

[bib32] Kutner, M. H., Nachtsheim, C. J., Neter, J., & Wasserman, W. (2004). *Applied linear regression models* (Vol. 4). New York, NY: McGraw-Hill.

[bib33] Ling, Y., & Hurlbert, A. (2004). Color and size interactions in a real 3D object similarity task. *Journal of Vision,* 4(9), 5, 10.1167/4.9.5. 15493966

[bib58] MacDonald, L. W., Biggam, C. P., Paramei, G. V. (Eds.), *Progress in colour studies: Cognition, language and beyond* (pp. 41–58). Amsterdam, NL: John Benjamins Publishing Company.

[bib34] Mollon, J. (1995). Seeing colour. In Lamb, T., Bourriau, J. (Eds.), *Colour: Art and science* (Vol. 7, pp. 127–151). Cambridge, UK: Cambridge University Press.

[bib39] Olkkonen, M., Hansen, T., & Gegenfurtner, K. R. (2008). Color appearance of familiar objects: Effects of object shape, texture, and illumination changes. *Journal of Vision,* 8(5), 13, 10.1167/8.5.13. 18842084

[bib40] Pitt, I. T., & Winter, L. M. (1974). Effect of surround on perceived saturation. *Journal of the Optical Society of America A,* 64(10), 1328–1331. 10.1364/josa.64.001328 4418539

[bib41] Regan, B. C., Julliot, C., Simmen, B., Viénot, F., Charles-Dominique, P., & Mollon, J. D. (2001). Fruits, foliage and the evolution of primate colour vision. *Philosophical Transactions of the Royal Society of London. Series B: Biological Sciences,* 356(1407), 229–283. 11316480 10.1098/rstb.2000.0773 PMC1088428

[bib42] Ronchi, L. R. (2002). On the dependence of brightness on target size. *Fondazione Giorgio Ronchi,* 79–95. ANNO LVII.N.1. 2002.

[bib43] Schiller, F., & Gegenfurtner, K. R. (2016). Perception of saturation in natural scenes. *Journal of the Optical Society of America A,* 33(3), A194–A206. 10.1364/JOSAA.33.00A194 26974924

[bib44] Schiller, F., Valsecchi, M., & Gegenfurtner, K. R. (2018). An evaluation of different measures of color saturation. *Vision Research,* 151, 117–134. 28551362 10.1016/j.visres.2017.04.012

[bib45] Sheather, S. (2009). *A modern approach to regression with R*. New York, NY: Springer Science & Business Media.

[bib46] Singer, B., & D'Zmura, M. (1994). Color contrast induction. *Vision Research,* 34(23), 3111–3126. 7975344 10.1016/0042-6989(94)90077-9

[bib47] Skelton, A., & Franklin, A. (2020). Infants look longer at colours that adults like when colours are highly saturated. *Psychonomic Bulletin & Review,* 27(1), 78–85. 31848908 10.3758/s13423-019-01688-5 PMC7000485

[bib48] Smith, P. H. (2016). Historians in the laboratory: Reconstruction of Renaissance art and technology in the Making and Knowing Project. *Art History,* 39(2), 210–233.

[bib49] Todd, J. T., & Norman, J. F. (2018). The visual perception of metal. *Journal of Vision,* 18(3), 1–17, 10.1167/18.3.9. 29677326

[bib51] Toscani, M., Valsecchi, M., & Gegenfurtner, K. R. (2013). Optimal sampling of visual information for lightness judgments. *Proceedings of the National Academy of Sciences,* 110(27), 11163–11168. 10.1073/pnas.1216954110 PMC3704015 23776251

[bib53] van Zuijlen, M. J., Lin, H., Bala, K., Pont, S. C., & Wijntjes, M. W. (2021). Materials In Paintings (MIP): An interdisciplinary dataset for perception, art history, and computer vision. *PLoS One,* 16(8), e0255109. 34437544 10.1371/journal.pone.0255109 PMC8389402

[bib54] van Zuijlen, M. J., Pont, S. C., & Wijntjes, M. W. (2020). Painterly depiction of material properties. *Journal of Vision,* 20(7), 1–17, 10.1167/jov.20.7.7. PMC7426625 32634227

[bib55] Wiebel, C. B., Toscani, M., & Gegenfurtner, K. R. (2015). Statistical correlates of perceived gloss in natural images. *Vision Research,* 115, 175–187. 25937518 10.1016/j.visres.2015.04.010

[bib56] Wiersma, L. (2020). ‘Colouring’—Material depiction in Flemish and Dutch baroque art theory. *Art & Perception,* 8(3–4), 243–265.

[bib57] Withouck, M., Smet, K. A., & Hanselaer, P. (2015). Brightness prediction of different sized unrelated self-luminous stimuli. *Optics Express,* 23(10), 13455–13466. 26074593 10.1364/OE.23.013455

[bib59] Xian, S. X., & Shevell, S. K. (2004). Changes in color appearance caused by perceptual grouping. *Visual Neuroscience,* 21(3), 383–388. 15518218 10.1017/s0952523804213062

[bib60] Zaidi, Q., Yoshimi, B., Flanigan, N., & Canova, A. (1992). Lateral interactions within color mechanism in simultaneous induced contrast. *Vision Research,* 32(9), 1695–1707. 1455741 10.1016/0042-6989(92)90162-c

